# An Overview of Managements in Meningiomas

**DOI:** 10.3389/fonc.2020.01523

**Published:** 2020-08-21

**Authors:** Lianhua Zhao, Wei Zhao, Yanwei Hou, Cuixia Wen, Jing Wang, Pei Wu, Zaiyu Guo

**Affiliations:** ^1^Department of Neurology, Tianjin TEDA Hospital, Tianjin, China; ^2^Department of Neurosurgery, Tianjin TEDA Hospital, Tianjin, China; ^3^Department of Radiotherapy, Xuzhou Central Hospital, Xuzhou, China; ^4^Department of Radiotherapy, Tianjin Medical University Cancer Institute and Hospital, Tianjin, China; ^5^Department of Neurosurgery, The First Affiliated Hospital of Harbin Medical University, Harbin, China

**Keywords:** meningioma, surgery, radiotherapy, stereotactic radiosurgery, target therapy

## Abstract

Meningioma is the most frequent primary tumor of the central nervous system. Important advances have been achieved in the treatment of meningioma in recent decades. Although most meningiomas are benign and have a good prognosis after surgery, clinicians often face challenges when the morphology of the tumor is complicated or the tumor is close to vital brain structures. At present, the longstanding treatment strategies of meningioma are mainly surgery and radiotherapy. The effectiveness of systemic therapy, such as chemotherapy or targeted therapy, has not been confirmed by big data series, and some clinical trials are still in progress. In this review, we summarize current treatment strategies and future research directions for meningiomas.

## Introduction

Meningioma is the most common central nervous system tumor originating from arachnoid cap cells. Meningioma account for about 30% of all primary intracranial tumors in adults, but are rare in children and adolescents (0.4–4.6%) (1). The total incidence of meningiomas is 83/100,000. Meningiomas are more common in women (female-biased sex ratio 2–4: 1) (2). The annual incidence of meningioma increased with age, from 0 to 19-years (0.14/100,000) to 75–84-years (37.75/100,000) (3). The median annual incidence of meningioma is lowest in African Americans (3.43 per 100,000 persons) and highest among Whites (9.52 per 100,000 persons) (4). However, the multivariate analysis results shows that African Americans are independent risk factors for relapse compared with Whites, Hispanics, and Asians (5).

Eighty to ninety percentage of meningiomas are benign (WHO grade I) and can be routinely followed up for the long term or cured by surgery and radiotherapy (2). The rest include atypical meningioma (WHO grade II) and anaplastic meningioma (WHO grade III or “malignant meningioma”), and the therapeutic effect is not satisfactory whether surgery, radiotherapy, or traditional chemotherapy is used.

**Figure 1:**
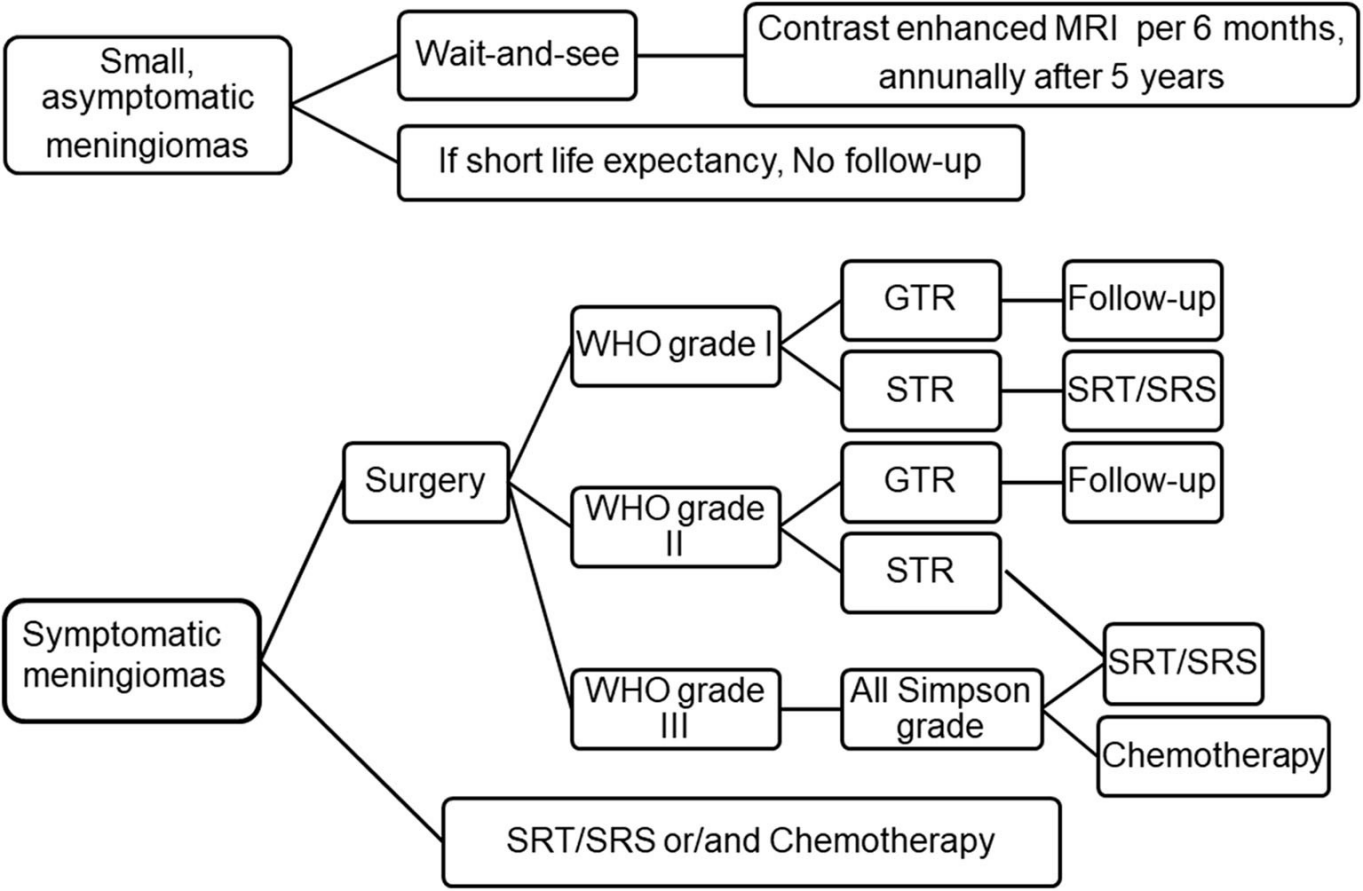
Current treatment strategies for meningioma. For small and asymptomatic meningiomas, an strategy of “wait and see” is recommended, clinical and MRI evaluation was performed every 6 months after an initial observation. If patients do remain asymptomatic, annually after 5 years. If the patient's life expectancy is short, follow-up may not be necessary. Symptomatic meningioma should be removed to the maximum extent. Patients who are unwilling to undergo surgery, the elderly or obviously disabled can choose SRT/SRS or chemotherapy. Patients with WHO grade I meningioma were followed up after GTR, and SRT/SRS was recommended after STR. For WHO grade II meningioma, intimate follow-up is recommended after GTR, while SRT/SRS is recommended after STR. For WHO grade III meningiomas, adjuvant radiotherapy are recommended regardless of the grade of resection. Adapted from Goldbrunner et al. (6). EANO guidelines for the diagnosis and treatment of meningiomas. WHO, world health organization; GTR, gross total resection; STR, subtotal resection; SRT, stereotactic radiotherapy; SRS, stereotactic radiosurgery.

The aim of this study is to review the current advancement of meningioma treatment. A comprehensive review has been made to collect all the articles related to meningioma treatment since 1993 until 2020. MEDLINE and PubMed database searches were performed. Related articles cited in the chosen studies were also investigated. We summarized the current treatment strategies of meningioma in the figure (Figure 1). Details of each treatment will be presented in the corresponding section.

## “Wait-and-See” Strategies

Small (tumor diameter ≤3 cm), asymptomatic (few or no symptoms or signs) meningiomas can be carefully observed and followed with regular Magnetic Resonance Imaging (MRI) scans. The approach is also applicable to old patients and patients with severe complications or poor physical conditions. The European Association of Neuro-Oncology (EANO) suggests that for asymptomatic or small meningioma, 6 months after the initial diagnosis, the dynamic changes in tumor should be evaluated with contrast enhanced MRI. If the patient remains asymptomatic, the patient is evaluated annually thereafter. After 5 years, this interval can be doubled. For patients with short life expectancy due to old age or severe complications, if the radiological diagnosis of benign meningioma is clear, follow-up is not required (6). However, if the tumor significantly enlarged or presents symptoms during follow-up period, active treatment is recommended. Tumor diameter ≥3 cm, peritumoral edema (PTE), age <60 years, lack of calcification, T2 hyperintense lesion are significantly correlated with the risk of symptom progression (7–11).

## Surgery

Surgical resection is the primary treatment choice for symptomatic meningiomas. The purpose of the operation is to relieve symptoms caused by the tumor, change the natural course of the tumor, and improve quality of life. The tumor should be removed surgically in patients with obvious mass effect and increased intracranial pressure. The factors that affect the surgical strategy are as follows: (1) surgical benefits; (2) surgical risks; (3) biological characteristics of tumor; (4) tumor mass effect or clinical symptoms; (5) subjective wishes of patients. Surgical risks were assessed based on the patient's general condition, tumor location, age, tumor size, and symptoms (2, 12). The location of the tumor is very important for the assessment of surgical risk. The surgical approach and resection of convex meningiomas are relatively simple and of low risk. The full exposure of surgical field and the careful separation of tumor capsule can protect the structure of artery and vein to the greatest extent, improve the success rate of operation of convex meningiomas, and reduce the disability rate. If the tumor is located in the olfactory sulcus, adjacent to the sagittal sinus, intraventricular, cerebellopontine angle, and falx cerebrum, the surgery has moderate risk. The removal of meningiomas involving the dural sinus, blood vessels, or cranial nerves is a great challenge for surgeons. The surgery for meningiomas originating from the clinoid process, cavernous sinus, and tuberculum sellae is of high risk (13). The petroclival area is the position where cranial nerves, cavernous sinus segment of internal carotid artery, basilar artery, superior cerebellar artery, and posterior cerebral artery converge. Tuberculum sellae meningiomas usually involve optic nerve and anterior cerebral artery complex. These complex structures often wrap around the surface of the tumor and adhere to the tumor tightly. Therefore, special attention should be taken during the operation.

Gross total resection (GTR) of meningiomas involving cortical veins or venous sinuses may damage the venous circulation. Subtotal resection (STR) can be performed when the venous sinuses are partially unobstructed (14). At present, it is generally recommended to resect the tumor outside the superior sagittal sinus. The residual tumor may recur. Imaging follow-up or adjuvant radiosurgery may be given for the residual tumor (15). For the tumors that invade the superior sagittal sinus without affecting the patency of the sinus, it is suggested that only the tumor outside the venous sinus be removed, and then the residual tumor in the venous sinus should be followed up regularly. It is recommended to resect the tumor after radiotherapy if the tumor is enlarged during the follow-up period. If the venous sinus has been completely occluded and the vein collateral circulation has been established, the occluded venous sinus can be removed by surgery after the detailed evaluation of these collateral veins, and these formed collateral veins should be protected during the operation. Traditional experience has shown that the risk of complete removal of the invaded sinus is not high and there is no need to reconstruct the venous circulation. Some scholars claim to reconstruct the venous circulation system on the basis of total tumor resection. The reconstruction of venous sinuses has potential benefits for patients with venous compensation affected or even patients with complete occlusion of venous sinuses. However, the safety and effectiveness have not been confirmed in multicenter randomized studies. The injury of unobstructed venous sinus may be followed by cerebral infarction, intracerebral hemorrhage, visual loss, infection, and other consequences. In our experience, total removal of meningiomas invading the venous sinus should not be the ultimate goal of surgery. No matter which operation method is chosen, the anatomy and compensation of the collateral vein and the invasion of the venous sinus must be clearly understood before the operation if we want to deal with the venous sinus during the operation.

Surgery microscope, neuronavigation technology, intraoperative neurophysiological monitoring, intraoperative imaging, adaptive hybrid surgery, and cavitational ultrasonic aspirators have greatly improved the success rate of surgery. The operation of skull base meningioma is challenging. Many skull base meningiomas cannot be completely resected even if the latest surgical methods are fully utilized. Endonasal approach can reach the ventral side of the deep skull base tumor, avoid the pulling of brain tissue during the operation, which is conducive to the safe resection of lesions, and even achieve Class Simpson I resection in some patients. The endonasal approach is more suitable for the removal of small meningiomas growing beside or below the optic chiasm. The endonasal approach are not suitable for large meningiomas, asymmetric meningiomas, or meningiomas which surround major vascellum and optic nerve. The narrow and limited operation space increases the risk of operation in the key anatomical position. The blood supply of skull base meningioma mainly comes from the ventral vessels of the tumor. The dura and its surface vessels in the basal region of the tumor can be exposed preferentially by endonasal approach. Endoscopic endonasal approach can be divided into standard endoscopic endonasal approach (SEEA) and expanded endoscopic endonasal approach (EEEA). EEEA can not only avoid pulling brain tissue, but also minimize the damage of optic nerve, reduce the congestion and edema of brain tissue, and maintain the integrity of appearance.

Meningiomas of skull base suitable for endoscopic treatment include olfactory sulcus meningioma, tuberculum sellae meningioma, petroclival meningioma, foramen magnum meningioma, etc. Cerebrospinal fluid leakage (9.5%), infection (5.4%), nerve injury (4.1%), vascular injury (2.7%) is a major complications of endoscopic endonasal approach (16). Endoscopic endonasal approach should be the primary choice for tuberculum sellae meningiomas with suspected involvement of the optic canal. The tuberculum sellae meningioma often grows into the optic canal through the medial edge of the cranial opening of the optic canal, which is the main reason for postoperative recurrence. Endoscopic treatment of tumors on the ventral side of the optic canal has natural anatomical advantages. However, whether transcranial approach or endoscopic endonasal approach should be used remains controversial (17). The biggest challenge of endonasal approach is the reconstruction of skull base, especially for the wide base meningioma. The incidence of cerebrospinal fluid leakage is as high as 30% (18). At present, it is considered that the multi-layer repair method of skull base reconstruction is more effective than the single-layer repair method, the tissue patch with blood supply is more beneficial than that without blood supply. The most commonly used patch is the self nasal septum mucosa flap with vascular pedicle, which can meet the needs of reconstruction of most skull base defects and reduce the incidence of cerebrospinal fluid leakage to <5% (19). The efficacy of endonasal approach depends on many factors, including the size, growth pattern, invasion degree, and transfer status of meningioma. Strict control of indications and contraindications of endonasal approach also has a certain impact on the prognosis of the operation. Endoscopic endonasal approach may be more suitable for small meningiomas located in midline anterior cranial fossa and may improve the visual impairment caused by tumors (20). However, if meningioma is too large, surrounded by blood vessels or calcified, endoscopic endonasal approach is not recommended (21). It is generally considered that the invasion of the medial side of the optic canal or the growth of tumor to the lateral part of the optic nerve is the contraindication of the endonasal approach. Endonasal approach should not be adopted when meningioma involves internal carotid artery, anterior cerebral artery, or anterior communicating artery. In order to maintain a clear field of vision, remove the lesion to the greatest extent, and avoid the damage of key nerves and blood vessels and adjacent anatomical structures in the operation area, the skull base bone should be removed as much as possible to open up a wide operation channel. The effect of surgery is also closely related to the professional skills of surgeons.

Up to date, there are several limited data comparing the effectiveness of endoscopic and microsurgery for meningiomas. Gaedner reported the combined use of endoscopy and microscopy in 35 cases of anterior skull base meningiomas (22). Devitiis reported the results of 51 cases of tuberculum sellae meningioma resected by transcranial approach and endoscopic surgery (23). The results of the two studies are consistent. Both believe that the early neurological complications of patients in the endoscopic endonasal approach group are lower, compared with traditional craniotomy, but the long-term recurrence rate and survival rate need to be further evaluated. More importantly, both reports indicate that the degree of resection is not affected by the approach, but by the patient's condition and tumor factors. Another study found that there was no significant difference between transcranial approach and endoscopic approach in perioperative mortality and incidence of GTR (24). The visual function of patients with tuberculum sellae meningioma improved more significantly after endoscopic surgery. However, the incidence of cerebrospinal fluid leakage after endoscopic surgery was higher than transcranial approach, olfactory groove meningiomas (25.1 vs. 10.5%) (25, 26) and tuberculum sellae meningiomas (19.3 vs. 5.81%) (17), which is almost three times of that of patients undergoing transcranial surgery. It is clear that further research is needed to determine the recurrence rate of these two methods, and with the development of endoscopy, it may be matched with craniotomy in terms of recurrence risk 1 day. We think that the choice of approach depends on the understanding of local anatomy and clinical experience of the surgeon. The imaging examination of the skull base structure before operation is helpful to know the size, location, blood supply, texture, adhesion status, and the adjacent structures such as nerves, blood vessels, and dura mater, which is very important for the choice of the approach. Combined approach, which combines the advantages of surgical microscope and endoscope, may be the future of meningioma surgery.

Meningiomas are usually resected to the maximal extent according to Simpson's criteria. The key point of the operation is to protect the normal brain tissue beside the tumor. It is difficult to completely remove tumors that are closely adhered to venous sinus or neurovascular tissue of cranial base without serious complications (27, 28). At present, STR is accepted by more and more neurosurgeons as a strategy to preserve the integrity of vein and nerve function (29).

Most meningiomas are rich in blood vessels. Selective vascular embolization is helpful to improve the GTR of skull base meningiomas, shorten the operation time, decrease the bleeding and reduce the incidence of postoperative complications. The vascular pedicle of many skull base meningiomas is located in the ventral side of the surgical approach, and the surgical channel is narrow and deep, which makes the surgical resection more difficult (30). Preoperative embolization can improve the safety of the operation and fully expose the tumor during the operation. Moreover, the ischemic necrosis and softening of tumor tissue caused by embolization can reduce the traction of peripheral nerve tissue during the operation. Therefore, vascular embolization may facilitate the completion of a surgical approach more safely. It can be used as a separate treatment for some patients who are not suitable for craniotomy, can slow down or prevent tumor growth, and can also be used as an adjuvant treatment before surgery. The safety and effectiveness of embolization alone for meningiomas have been questioned (31). For meningiomas with multiple blood supply, it is not recommended to embolize all the blood supply arteries, embolization of the primary artery is an appropriate choice. The complication of cerebral infarction is easy to appear in internal carotid artery branch embolism (32). For large meningiomas, meningiomas with blood supply mainly from the branches of the external carotid artery, or meningiomas located in difficult surgical sites with abundant blood supply, the maximum benefit may be achieved from preoperative selective embolization (31). Preoperative embolization is suggested in the following situations: complicated blood supply vessels, severe PTE affecting the identification of tumor boundary, tumor proximity to functional areas, and the dural sinus, scalp, and skull are involved (33). With the progress of interventional therapy techniques, the risk of preoperative embolization has decreased year by year. Studies have shown that the complication rate of preoperative embolization is only 2.6–12% (34, 35). Severe neurological dysfunction after preoperative embolization of meningiomas includes occlusion of distal vessels, reflux of embolic materials, bleeding, and swelling of tumors caused by occlusion of blood vessels. Therefore, the potential benefits and adverse consequences of embolization must be carefully evaluated before embolization. The incidence of hemorrhagic complications after vascular embolization is higher than that of ischemic complications (36). The deep infiltration of embolic particles and the necrosis caused by blood flow blocking make the tumor easy to bleed (36). The dissolution of granules and the remission of vasospasm may lead to ineffective reperfusion of vascular bed and aggravate the edema and swelling of tumor. Therefore, the intracranial mass effect caused by very large meningiomas may be aggravated after embolization. It is reported that the interval time between embolization and surgery ranges from 1 day to more than 1 week (37). Extending the interval between embolization and surgery may maximize the benefit of embolization. Some experts suggested that the best time for operation is 7–9 days after embolization (38). However, recanalization or collateral circulation may occur at more than 1 week after embolization. Therefore, most centers perform surgery within 7 days (37).

5-aminolevulinic acid (5-ALA) is an indirect fluorophore, which can be absorbed by tumor and converted into a fluorescent substance protoporphyrin IX (PP IX). The surgeon can directly see the fluorescence of tumor through fluorescence microscope and other equipment. 5-ALA can calibrate cancer cells, make them fluoresce, and can improve tumor resection rate. It has been applied to different types of central nervous system tumors, including ependymoma, hemangioblastoma, metastatic brain tumor, and intracranial meningioma. 5-ALA fluorescence guided tumor resection has been proved to be one of the effective methods to improve the “gross resection rate” of high-grade gliomas (39). However, the influence of histopathological grading and previous treatment on the fluorescence ability of meningioma cells remians still unclear. The effectiveness of 5-ALA mediated Fluorescence-guided surgery for meningiomas a needs further evaluation in the future (40, 41).

The postoperative complications of meningioma include cerebral hemorrhage, infection, neurological deficit, brain edema, epilepsy, etc. The incidence of postoperative intracranial hemorrhage is about 2.6%. The mechanism includes abnormal coagulation function, small vessel injuries caused by excessive pulling of brain tissue, bleeding of surgical wound, blood pressure fluctuation post operation, or other potential diseases. The incidence of postoperative infection was 2.7%. The location of the tumor is a predictor of postoperative infection, the incidence of infection in skull base meningiomas is four times higher than that in non-skull base meningiomas. Prolonged operation time is also associated with an increased risk of infection (42). Normative surgery practice, adequate rinsing of surgical site, and prophylactic application of antibiotics can reduce the postoperative infection rate. The incidence of postoperative neurological deficits directly related to surgery is 2–30%, which depends on the location and resection range of the tumor. Meningiomas in non-functional areas are usually completely removed with minimal complications. Surgery of cranial base meningiomas may injure the cranial nerve. When the tumor invades the venous sinus, surgery may accidentally injure the superior sagittal sinus and the diploic veins, resulting in postoperative venous infarction. PTE can be seen in about 46–92% of meningiomas in different degrees. PTE can cause clinical symptoms and complicate surgery, which is closely related to poor prognosis after surgery. Preoperative PTE may be a risk indicator for poor prognosis of the elderly (43). PTE is caused by tumor compression, tumor features such as invasiveness, high histological grade, histopathology as secretory type, microcystic type and/or hemangioma type, and high expression of vascular endothelial growth factor (VEGF). Corticosterone steroid hormone is the predominant drug for the treatment of PTE. Anti-angiogenic therapy (e.g., bevacizumab) may be considered in case of poor hormone effect (44). Studies have shown that early postoperative hyperbaric oxygen therapy can significantly reduce PTE, improve Karnofsky Performance Score (KPS), and reduce the incidence of neurological dysfunction (45).

In patients with meningiomas, the rate of new seizure after surgery is about 12–19% (46). Epilepsy after meningiomas surgery may be related to meningiomas themselves or craniotomy. It has demonstrated that maximum diameter >1 cm of PTE, WHO grade II and III tumors and low-range resection (Simpson grades III-v) are independent predictors of postoperative poor seizure outcomes (47). Preventive application of antiepileptic therapy remains controversial. A recent meta-analysis shows that preventive use of anti-epileptic drugs is ineffective for meningiomas patients who have no previous history of epilepsy (48). The American Academy of Neurology recommends that patients with no previous history of epilepsy should stop prophylactic antiepileptic therapy 1 week after surgery (49). Reducing brain tissue or vascular injury during operation can reduce postoperative neurological deficits and improve seizures (50). Whether postoperative epilepsy is related to tumor STR remains controversial. Non-enzyme-induced antiepileptic drugs are recommended for patients who have experienced one or more meningioma-related seizures. Levetiracetam and gabapentin have good efficacy and tolerance for patients with persistent epilepsy.

In addition, MR-guided laser ablation therapy (MR-LITT) is one of the most promising minimally invasive surgical techniques. MR-LITT can accurately ablate meningiomas lesions and avoid damage to surrounding tissues. For patients with PTE symptoms, LITT may be a feasible alternative therapy if drug therapy is not good enough (51). However, these effects still need further randomized controlled studies to confirm.

## Radiation Therapy

Radiation therapy (RT) is suitable for the following patients: patients diagnosed with WHO grade II or grade III meningioma; patients after STR; patients who have lost the opportunity of surgery for various reasons or have a recurrence and are not suitable for resection (52). The purpose of radiotherapy is to reduce its proliferation ability and control its progress. Fractionated radiotherapy increases the tolerance dose of important intracranial structures (such as visual pathways) and reduces the side effects of radiotherapy as much as possible. Conventional fractionated radiotherapy for STR postoperative and recurrent meningiomas can significantly improve the local tumor control rate. Unconventional fractionated radiotherapy includes hypofractionated radiotherapy and Hyperfractionated radiotherapy. There are few studies on hyperfractionated radiotherapy in the treatment of meningiomas.

With the development of computer technology, radiotherapy is more accurate and individualized. Precision radiotherapy technology includes three-dimensional conformal radiotherapy (3D-CRT), intensity modulated radiotherapy (IMRT), Image guided radiotherapy (IGRT), real-time dynamic radiotherapy, etc. Stereotactic radiotherapy (SRT) is an improvement of conformal radiotherapy. SRT technology can irradiate a specific target with a large dose once, the attenuation of radiation dose outside the target area is steep, and normal tissues around the focus are not damaged. Fractionated stereotactic radiotherapy (FSRT) can reduce the exposure dose of peripheral normal brain tissue in high dose radiation. Compared with conventional radiotherapy, FSRT has similar therapeutic results. Stereotactic radiosurgery (SRS) was developed by combining radiotherapy and stereotactic. SRS is suitable for meningiomas with a maximum diameter of <3 cm and located more than 3 mm from radiosensitive structures such as optic nerve (53). Early SRS devices used only a single fractionated therapy. Current radiosurgery devices can use frameless radiosurgery techniques, allowing repeated fractionated therapy or large fractionated radiosurgery (54).

The recurrence rate of WHO grade I meningioma after GTR is relatively less, and most experts advocate that postoperative adjuvant radiotherapy is not required. However, WHO grade I meningioma has a high recurrence rate after STR surgery. Radiotherapy is recommended if salvage total resection is not possible in the future. Grade II and III meningiomas are invasive tumors. Even after obvious Simpson I resection, the risk of recurrence is still high, reaching 30–40% and 50–80%, respectively, after 5 years (10). Therefore, in the initial treatment, surgery is often combined with radiotherapy. Radiotherapy for WHO II meningiomas remains controversial, and trials are currently underway to confirm the role of postoperative radiotherapy for completely resected WHO II meningiomas (6). For WHO III meningiomas, routine radiotherapy is recommended after surgery regardless of the surgical method. Our point of view is the potential benefits of “radiotherapy” need to be carefully weighed against the side effects of “radiotherapy” after total atypical meningioma resection. For atypical meningioma patients with STR, we recommend “radiotherapy” rather than observation. Metastasis of meningiomas is rare, if the number of metastatic meningiomas is too large to be removed completely, or the patient's physical condition is not suitable for surgery, fractionated radiotherapy can be used (55).

Tanzler et al. (56) reported that PFS of primary radiotherapy for patients with grade I meningioma for 5 and 10 years was 99% (postoperative RT was 96 and 93%). Santacroce et al. (57) reported a PFS incidence rate of 92.7% in 10 years after nearly 3,000 meningioma patients received radiotherapy alone without surgery. Pollock et al. (58) found no difference between SRS and GTR in 7-year PFS rate (both >95%). Kokubo reported a 5-year local control rate of 41% for benign meningiomas and 30% for atypical or malignant recurrent meningiomas (59). It is not clear whether the PFS after radiotherapy is related to previous surgery. In a retrospective observational study, the PFS in the radiotherapy group is superior to Simpson's 2–5 stage resections when comparing surgical resection and radiotherapy for meningiomas smaller than 35 mm in diameter.

Metellus et al. (60) reported the long-term follow-up results of 53 cases with cavernous sinus meningiomas who received conventional fractionated 3D-CRT. Twenty-eight cases (52.8%) were treated with radiotherapy alone, 25 cases (47.2%) were treated with postoperative adjuvant therapy. The average follow-up time was 6.9 years. PFS rates in 5 and 10 years were 98.1%, 95.8%, 31 cases (58.5%) were improved in clinical symptoms, 20 cases (37.7%) were stable in symptoms, 3 cases (57%) had acute radiation reaction, and 1 case (19%) had late injury. Hemmati et al. analyzed 99 patients with atypical meningioma (WHO grade II), of which 19 patients received IMRT after tumor resection and the remaining 80 patients only underwent surgical resection. The median follow-up period was 37 months. The results showed that the median PFS of patients receiving IMRT was significantly longer than that of the simple operation group (64 vs. 37 m) (61).

A retrospective study of 5,300 meningioma patients from 15 centers showed that the PFS rates of SRS in 5 and 10 years were 95.2–97% and 88.6–94%, respectively (62), and the complication rate was 6.6% (57). A review shows that the 5-year rates of gamma-knife SRS, LINAC SRS, and FRT PFS are 93.6,95.6, and 97.4%, respectively (*P* = 0.32). SRS is twice higher than FRT in tumor volume reduction rate, tumor recurrence or progression rate is 3–5.8%, and there is no statistical difference between the two methods (*p* > 0.05) (63). WHO grade and previous radiotherapy history are reliable long-term predictors of overall prognosis of gamma -knife SRS therapy (64). The overall 5-year control rate of WHO grade I meningioma patients receiving gamma knife adjuvant therapy was 93%. The total PFS rate after STR followed with SRS seems to be equivalent to GTR (65). The tumor control rates of adjuvant SRS therapy for WHO grade II and III tumors are 50 and 17%, respectively (66). For STR meningioma (Simpson Grade II-IV), the 3 and 7-year PFS rate of SRS were better than surgery (58). Adjuvant radiotherapy can improve the long-term control and overall survival of WHO grade III meningiomas. PFS increased from 28% of GTR to 57% of GTR followed by adjuvant radiotherapy at 5 years. Aghi et al. (67) described that 8 atypical meningioma patients (108 in total) did not relapse after receiving GTR plus radiotherapy, while the relapse rate of GTR alone was 30% (average follow-up 3 years).

Factors affecting the effect of SRS on meningiomas include WHO classification of tumor, tumor location and size, patient age, time interval between SRS, and initial tumor resection and radiation dose, etc. (68). Tumor volume >8 cm^3^ is the most important factor for poor prognosis of benign meningiomas treated by SRS (69). The improvement of clinical symptoms in non-single-session gamma knife radiosurgery (non-SS GKS) patients may be twice as much as that in single-session gamma knife radiosurgery (SS GKS) patients. However, with the increase of SRS treatment volume for high-grade meningiomas, the incidence of radiotherapy-related complications increases (5–23%) (70). The most common adverse reactions were epilepsy (12.0%) (71), cranial nerve injury (5.5%), and PTE (5.3%) (72).

The timing and method of radiotherapy are still controversial. There was no difference in overall survival in patients with STR or STR plus radiotherapy. It is safe to wait for the disease to progress before radiotherapy (73). At present, there is no data showing that radiotherapy timing will affect the long-term survival rate. It is suggested that small asymptomatic meningiomas can be observed first, and radiotherapy should be performed if tumor progresses. For benign meningiomas invading venous sinus, it is necessary to weigh early radiotherapy, surgical resection and observation. It is not clear whether SRT or SRS should be used for atypical meningiomas (AM) (74). There are many factors that determine SRS or SRT in the treatment of meningiomas. Physical factors (tumor size, margin, optimal dose), biological factors (histology of metastatic tumor, use of systemic drugs) and clinical factors (life expectancy, complications) all play a role in decision-making (75). A study found that in 50 patients with atypical meningiomas, the average follow-up time was 86 months. Twenty-one patients (42%) received SRS. The local control rates of tumor for 2 and 5 years were 91 and 88% respectively. Twenty-nine patients (58%) received SRT. The local control rates of tumor for 2 and 5 years were 71 and 69%, respectively. There was no significant difference between SRS and SRT.

Compared with photon radiation therapy, proton radiation therapy, and neutron radiation therapy can irradiate the target more accurately and greatly reduce the radiation toxicity to surrounding normal tissues, but they are still in the development stage.

## Chemotherapy

**Figure 2:**
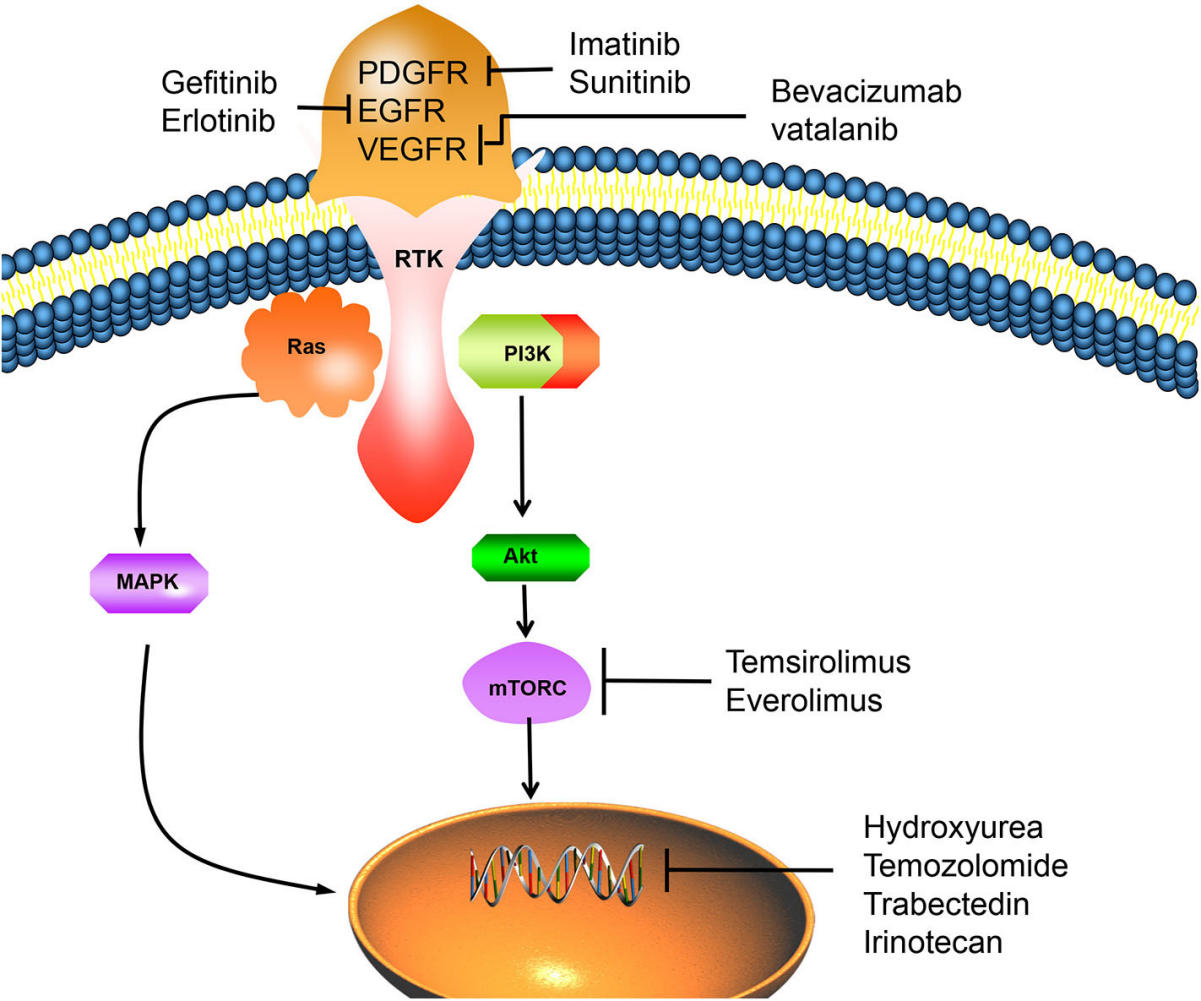
The overexpression of RTK can activate important mitogenic pathways, including Ras, MAPK, PI3K-Akt, Mtor, and other intracellular signals, which can promote the proliferation of tumor cells. However, PDGFR/EGFR/VEGFR inhibitors can inhibit the activation of RTK, thus reverse this process and lead to tumor cell apoptosis. Chemotherapy drugs such as hydroxyureae and temozolomide can act on cell nucleus, inhibit tumor cells proliferation by inducing cell apoptosis. PDGFR, platelet-derived growth factor receptor; EGFR, epidermal growth factor receptor; VEGFR, vascular endothelial growth factor receptor; RTK, receptor tyrosinekinase; Ras, PI3K, phosphatidylinositol 3-kinase; MAPK, mitogen activated protein kinase; Akt, protein kinase B; mTORC, mammalian target of rapamycin C.

Drug therapy can only be carried out when surgery and radiotherapy strategies are no longer available, such as recurrent or progressive meningiomas. There are a variety of chemotherapy drugs and molecular targeted drugs for the treatment of non-benign meningiomas, such as alkylating agents, tyrosine kinase inhibitors, endocrine drugs, interferon, targeted molecular pathway inhibitors, etc. (Figure 2). Although many drugs have shown efficacy in preclinical studies and some clinical applications, there is no consistently effective drug found in different clinical studies (76).

Hydroxyurea (HU) is a ribonucleic acid reductase inhibitor, which induces apoptosis of meningiomas cells by preventing the growth of S phase of cell cycle. HU has been used as adjuvant therapy for meningiomas that have not been completely resected or recurred. Weston et al. found that although HU may prevent some patients from progressing, it does not reduce the tumor size and causes significant side effects (77). Chamberlain published a retrospective case series study. This study retrospectively analyzed 35 patients with high-grade meningiomas who relapsed after surgery and radiotherapy (WHO Grade II, 22 cases; WHO Grade III, 13 cases), the total PFS rate at 6 months was 3%, and the median PFS was only 2.0 months (78). It shows that HU has very limited activity although it is well-tolerated.

Temozolomide (TMZ), an alkylating agent, failed to prolong PFS of recurrent meningiomas. Chamberlain et al. (79) treated 16 patients with refractory meningiomas with temozolomide. Tumor progression time was 2.5–5.0 months (median 5.0 months). The survival time ranged from 4 to 9 months (median 7.5 months). Irinotecan is a topoisomerase I inhibitor, which can cause DNA double strand breaks. A pre-clinical study found that irinotecan can inhibit the growth of meningeal cells (80). However, the subsequent Phase II trial failed to prove its clinical efficacy.

It has been reported that recombinant interferon α-2b is effective for a few malignant meningiomas patients (81). A study observed the therapeutic effect of interferon on 35 patients with recurrent WHO grade I meningiomas. PFS rate were 54 and 31% at 6 and 12 months, respectively, and the median progression time was 7 months, suggesting that interferon is an effective drug for the treatment of recurrent low-grade meningiomas (82). However, other studies have not reached a consistent conclusion.

Genomics studies have confirmed the importance of mutations such as NF2, TRAF7, KLF4, AKT1, SMO, PI3KCA, and POLR2A in the occurrence and development of meningiomas (83). Fifty to sixty percentage of meningiomas patients have mutation of tumor suppressor gene neurofibromatosis type 2 (NF2) (84). The NF2 gene product merlin is a tumor suppressor and mediates inhibition of cell proliferation (85). Gene mutations drive key mitogenic pathways, including mitogen-activated protein kinase (MAPK), phosphoinositide 3-kinase (AKT), mechanistic target of rapamycin (mTOR), extracellular signal-regulated kinase (ERK), etc. (86). Gene mutation can also overexpress receptor tyrosine kinases (RTKs), so more and more receptor tyrosine kinase inhibitors are used in targeted therapy (87).

The high expression of platelet-derived growth factor receptor (PDGFR) is closely related to the development of malignant meningiomas and atypical meningiomas. Imatinib combined with HU was used to treat recurrent or invasive meningiomas. Of the 21 patients receiving combined therapy, 67% had no imaging progress. The results showed that imatinib combined with HU was well-tolerated, but had little effect on grade II or III meningiomas (88). Sunitinib is a small molecule tyrosine kinase inhibitor targeting vascular endothelial growth factor receptor and PDGF. A prospective, multicenter, single-arm phase II clinical trial of sunitinib in the treatment of malignant meningiomas showed that 42% of patients achieved no tumor progression within 6 months. MR perfusion imaging confirms that sunitinib can reach the location of the lesion and play a role in the vascular system of the tumor, however, further study is needed to confirm whether these effects can produce beneficial clinical effects (89).

Over-expression of epidermal growth factor receptor (EGFR) is found in over 60% of meningiomas (90). Receptor activation of epidermal growth factor (EGF) or transforming growth factor-a (TGF-a) can promote *in vitro* proliferation of meningiomas cells (91). In a study of 25 patients with recurrent meningiomas treated with the EGFR inhibitors Gefitinib and Erlotinib. Although the treatment is well-tolerated, neither gefitinib nor erlotinib has no obvious activity on recurrent meningiomas. It suggests that EGFR alone may not be a valuable therapeutic target. Therefore, it is necessary to evaluate the combined application of multi-target inhibitors and EGFR inhibitors with other targeted molecular agents (76, 92).

VEGF was found expression in 84% of meningiomas, and VEGF receptor (VEGFR) was expressed in 67% of meningiomas (93). The expression level of VEGF and VEGFR in meningiomas increases with the increase of tumor grade. Inhibition of VEGF and VEGFR may have significant anti-tumor effect. Studies have shown that bevacizumab, a VEGF inhibitor, has clinical benefits in meningiomas with no response to surgery or radiotherapy, and can improve the survival rate of patients (94). However, due to the lack of strong clinical evidence for improving survival rate and related toxicity, the treatment of meningiomas with bevacizumab should be carefully evaluated. An ideal randomized controlled trial is needed to better determine the effect of this drug in the treatment of meningiomas (95). Vatalanib can effectively inhibit VEGFR and PDGFR and has anti-tumor activity in grade II and III meningiomas (87).

mTORC1 can attenuate RTK signals through PI3K and Akt pathway, thus forming a negative feedback loop. Inhibitors of mTOR pathway such as Temsirolimus and Everolimus have been proved to be effective in preventing meningiomas progression (96, 97). In addition, *in vitro* studies have demonstrated that retinol-like compounds such as Fenretinide can bind to the retinoic acid receptor (RAR) to induce apoptosis in meningiomas cells (98). Clinical trials of Vismodegib and Afureserib, specific drugs for meningiomas with mutations in SMO and AKTl genes, are under way. This trial is the first to target a specific mutant meningioma, and the results remain to be seen (99).

Studies have shown that there is a strong relationship between sex hormones and meningiomas. Estrogen receptor (ERs) is expressed at a low level in about 10% of meningiomas patients, while progesterone (PRs) and androgen receptor are expressed in 70% of meningiomas patients (100). Due to the low expression level of ERs, the treatment of ERs antagonist tamoxifen has not shown any effective results. The results of antiprogestin mifepristone study are also mixed (101). There have been no reports of androgen receptor antagonists in meningiomas. Somatostatin (SST) plays an important role in regulating the proliferation of normal cells and tumor cells. Long half-life SST analogs are now recommended for systemic treatment of unresectable or radiorefractory relapsed meningiomas (102). A recent study analyzed the efficacy of everolimus and octreotide in the treatment of recurrent meningiomas, and the results showed that the overall PFS6 was 55%. The 6 and 12-month survival rates were 90 and 75%, respectively. After 3 months of treatment, the growth rate of 78% tumor volume decreased significantly, that is the decrease was more than 50%. The study suggests that the combination of everolimus and octreotide has better anti-meningioma activity (103).

## Gene Therapy

Gene therapy is the introduction of genetic material (DNA or RNA) into human cells to correct or compensate for gene defects and abnormalities in order to achieve therapeutic purposes. Researchers found that adenovirus virus and herpes virus can be effectively transduced into meningiomas cells. Herpes simplex virus is the first oncolytic virus effective in treating meningiomas (104). Due to the short duration of therapeutic effect and uncontrollable insertion mutation, only a few preclinical studies have been reported, which also provides a new direction for gene therapy of meningiomas.

## Prognosis and Recurrence

The most reliable prognostic factors for meningiomas are histological grade (WHO grade) and resection degree (Simpson grade) (105). Meningiomas are mostly benign. The results of surgical treatment vary with the location and treatment of meningiomas. The tumors located in the medial sphenoid ridge, cavernous sinus, and clivus have poor prognosis, high operative mortality, many postoperative sequelae and poor quality of life (106).

Recurrence rate of meningiomas after operation is usually between 13 and 40%. Recurrence rate of meningiomas has a great correlation with Simpson classification degree of resection (10). The recurrence rate of Simpson grade I surgery patients is 9%, grade II is 19%, and grade III is 29%. Postoperative patients should receive regular imaging examination due to the recurrence rate of meningiomas also increases with the extension of follow-up time. After STR of the lesion (Simpson IV grade), almost all patients relapsed after more than 15 years of followed up, of which 60% died, and most occurred within 10 years.

Robert Sumkovski et al. found that sex, age, Karnofsky score etc. have predictive value for recurrence of different types of meningiomas (107). Histological grading of meningiomas also affects its recurrence, and with the increase of pathological grading, the recurrence rate increases greatly. The recurrence rate of WHO grade I meningiomas is 7–23%, WHO grade II meningiomas is 50–55%, and WHO grade III meningiomas is 72–78% in 5 years after total resection (70). The gene distribution of meningiomas varies with tumor location and may also affect prognosis. When recurrent meningiomas have symptoms, surgery should be considered first, and SRS/RT adjuvant therapy should be given after surgery. In the 16 patients with recurrent meningioma treated by radiotherapy, the disease-free survival rate was 78%, compared with only 11% for those treated with surgery alone (108).

## Outlook

With the continuous progress of skull base surgery, anesthesia technique, MR, neurovascular reconstruction and ultrasound, the GTR of meningiomas, and the prognosis of the patients have been greatly improved. Patients with meningiomas should be treated individually in multiple disciplines, modes and stages, and tumors should be removed and controlled to the greatest extent on the basis of ensuring the cranial nerve function and quality of life of patients. Stereotactic techniques, including gamma knife, linear accelerator, and proton beam radiotherapy, enable meningiomas to be treated with radiotherapy while preserving important nerve structures. Chemotherapy, targeted therapy, and immunotherapy for meningiomas are also under exploration. DNA methylation is closely related to tumors, and its characteristics can provide important basis for individualized treatment of different subtypes of meningiomas (109). Lymphocyte telomere length (LTL) is significantly correlated with increased risk of meningiomas (110). These studies may explain the causes of the occurrence and progression of brain tumor lesions in the future, thus enriching the treatment methods for meningiomas at all levels and bringing better prognosis to patients.

## Author Contributions

WZ and LZ collected, analyzed the clinical data, and wrote part of the manuscript. ZG, JW, YH, and CW participated in the data analysis and interpretation. PW worked on the manuscript revision. All authors read and approved the final manuscript.

## Conflict of Interest

The authors declare that the research was conducted in the absence of any commercial or financial relationships that could be construed as a potential conflict of interest.
