# Corrigendum: An Overview of Managements in Meningiomas

**DOI:** 10.3389/fonc.2020.599431

**Published:** 2020-09-24

**Authors:** Lianhua Zhao, Wei Zhao, Yanwei Hou, Cuixia Wen, Jing Wang, Pei Wu, Zaiyu Guo

**Affiliations:** ^1^Department of Neurology, Tianjin TEDA Hospital, Tianjin, China; ^2^Department of Neurosurgery, Tianjin TEDA Hospital, Tianjin, China; ^3^Department of Radiotherapy, Xuzhou Central Hospital, Xuzhou, China; ^4^Department of Radiotherapy, Tianjin Medical University Cancer Institute and Hospital, Tianjin, China; ^5^Department of Neurosurgery, The First Affiliated Hospital of Harbin Medical University, Harbin, China

**Keywords:** meningioma, surgery, radiotherapy, stereotactic radiosurgery, target therapy

In the original article, there were mistakes in the order of Figure 1 and Figure 2 as published. The positions of the two figures were reversed. The corrected Figure 1 and Figure 2 appear below.

**Figure 1:**
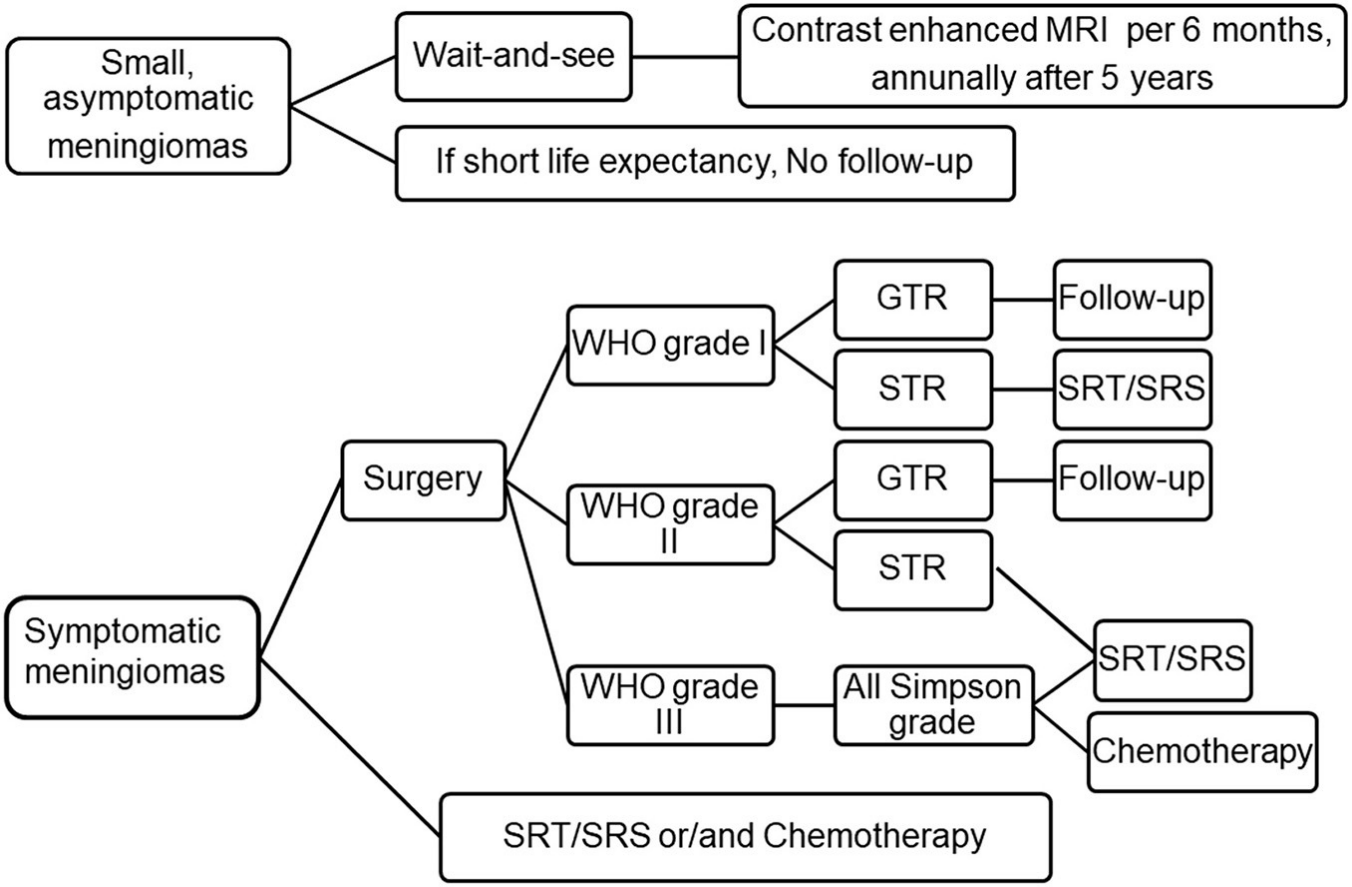
Current treatment strategies for meningioma. For small and asymptomatic meningiomas, an strategy of “wait and see” is recommended, clinical and MRI evaluation was performed every 6 months after an initial observation. If patients do remain asymptomatic, annually after 5 years. If the patient's life expectancy is short, follow-up may not be necessary. Symptomatic meningioma should be removed to the maximum extent. Patients who are unwilling to undergo surgery, the elderly or obviously disabled can choose SRT/SRS or chemotherapy. Patients with WHO grade I meningioma were followed up after GTR, and SRT/SRS was recommended after STR. For WHO grade II meningioma, intimate follow-up is recommended after GTR, while SRT/SRS is recommended after STR. For WHO grade III meningiomas, adjuvant radiotherapy are recommended regardless of the grade of resection. Adapted from Goldbrunner et al. (6). EANO guidelines for the diagnosis and treatment of meningiomas. WHO, world health organization; GTR, gross total resection; STR, subtotal resection; SRT, stereotactic radiotherapy; SRS, stereotactic radiosurgery.

**Figure 2:**
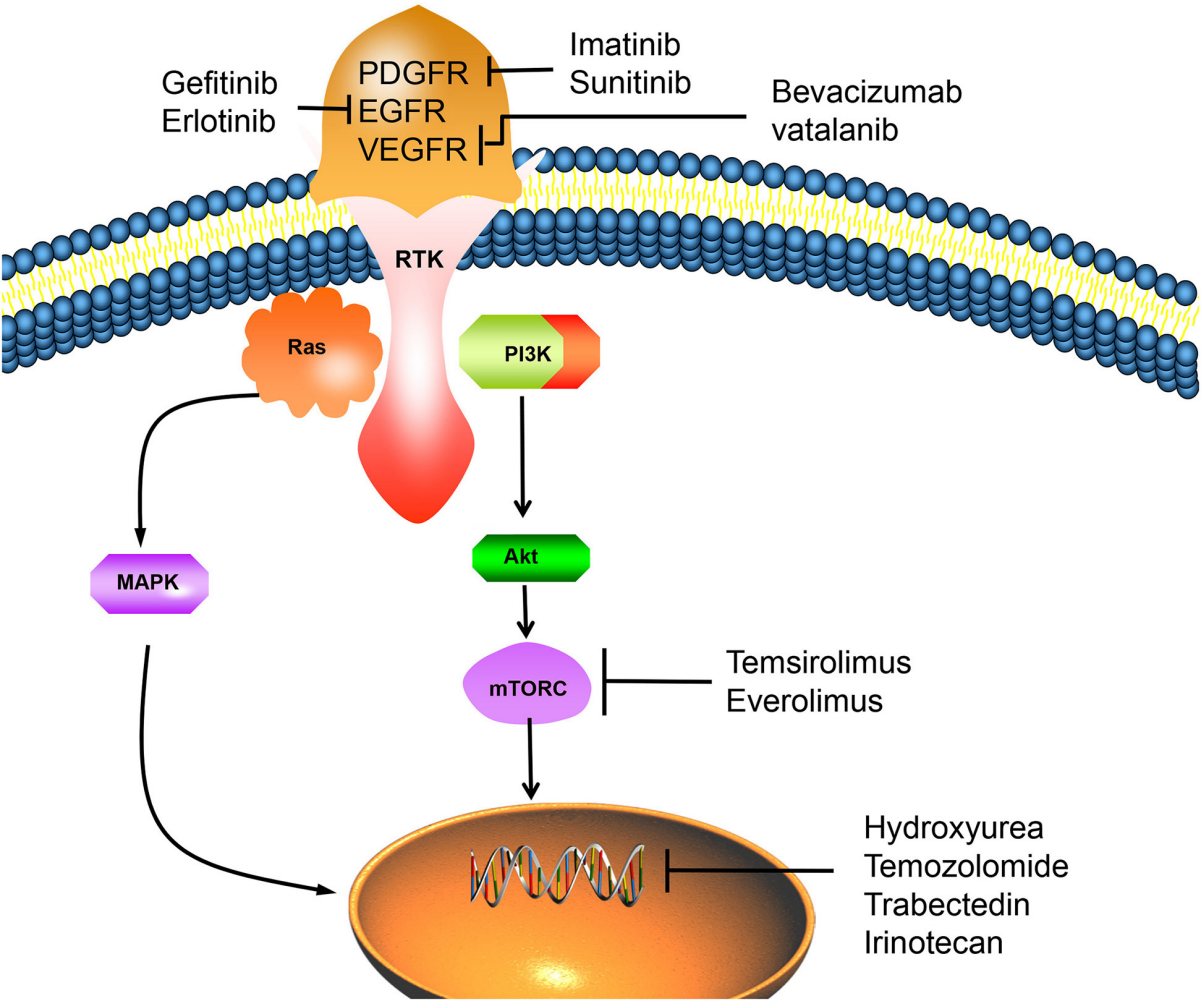
The overexpression of RTK can activate important mitogenic pathways, including Ras, MAPK, PI3K-Akt, Mtor, and other intracellular signals, which can promote the proliferation of tumor cells. However, PDGFR/EGFR/VEGFR inhibitors can inhibit the activation of RTK, thus reverse this process and lead to tumor cell apoptosis. Chemotherapy drugs such as hydroxyureae and temozolomide can act on cell nucleus, inhibit tumor cells proliferation by inducing cell apoptosis. PDGFR, platelet-derived growth factor receptor; EGFR, epidermal growth factor receptor; VEGFR, vascular endothelial growth factor receptor; RTK, receptor tyrosinekinase; Ras, PI3K, phosphatidylinositol 3-kinase; MAPK, mitogen activated protein kinase; Akt, protein kinase B; mTORC, mammalian target of rapamycin C.

The authors apologize for this error and state that this does not change the scientific conclusions of the article in any way. The original article has been updated.

